# Divalent Europium-containing colloidal metal halide nanocrystals for light-emitting applications

**DOI:** 10.1186/s40580-025-00496-z

**Published:** 2025-06-29

**Authors:** Ho Young Woo, Mi Yeon Yu, Seung Hyeon Kim, Da Won Lee, Yoonjoo Choi, Yerin Kim, Giyong Park, Hyungyoon Choi, Taejong Paik

**Affiliations:** 1 School of Integrative Engineering, Chung-Ang University, Seoul, 06974 Republic of Korea; 2 Department of Intelligent Semiconductor Engineering, Chung-Ang University, Seoul, 06974 Republic of Korea

**Keywords:** Nanocrystal, Nanoparticle, Europium, Luminescence, Light-emitting diode

## Abstract

Lanthanide-based inorganic nanomaterials have been widely utilized as luminescent materials for broad-ranging applications in lighting, display, and optoelectronic devices. Among lanthanide elements, divalent europium (Eu^2+^) has recently gained significant attention owing to its excellent photoluminescence (PL) properties, such as a short radiative decay lifetime, narrow PL bandwidth, and wide emission range from ultraviolet to near-infrared. Particularly, colloidal metal halide nanocrystals (MHNCs) offer unique advantages as inorganic hosts for Eu^2+^ owing to their excellent phase purity, chemical and optical stability, and colloidal stability for facile integration via solution processes. In addition, the PL properties of Eu^2+^, originating from the parity-allowed 4f–5d transitions, can be precisely controlled by tuning the phase and compositions of MHNCs. Therefore, an in-depth understanding of the Eu^2+^ PL mechanism and synthesis of phase-pure MHNCs is essential for the advancement of Eu^2+^-based MHNCs as novel emitters. This review summarizes recent developments in Eu^2+^-based colloidal MHNCs and their PL properties. First, the local factors affecting the luminescence properties of Eu^2+^ in inorganic hosts are discussed. Subsequently, recent advances in the synthesis of Eu^2+^-based MHNCs using different host–dopant frameworks, their optical proprieties, and applications are outlined. This comprehensive review provides valuable insights for designing high-performance emitters, particularly for achieving deep-blue emission in light-emitting diodes and high-energy scintillators.

**Graphic Abstract:**

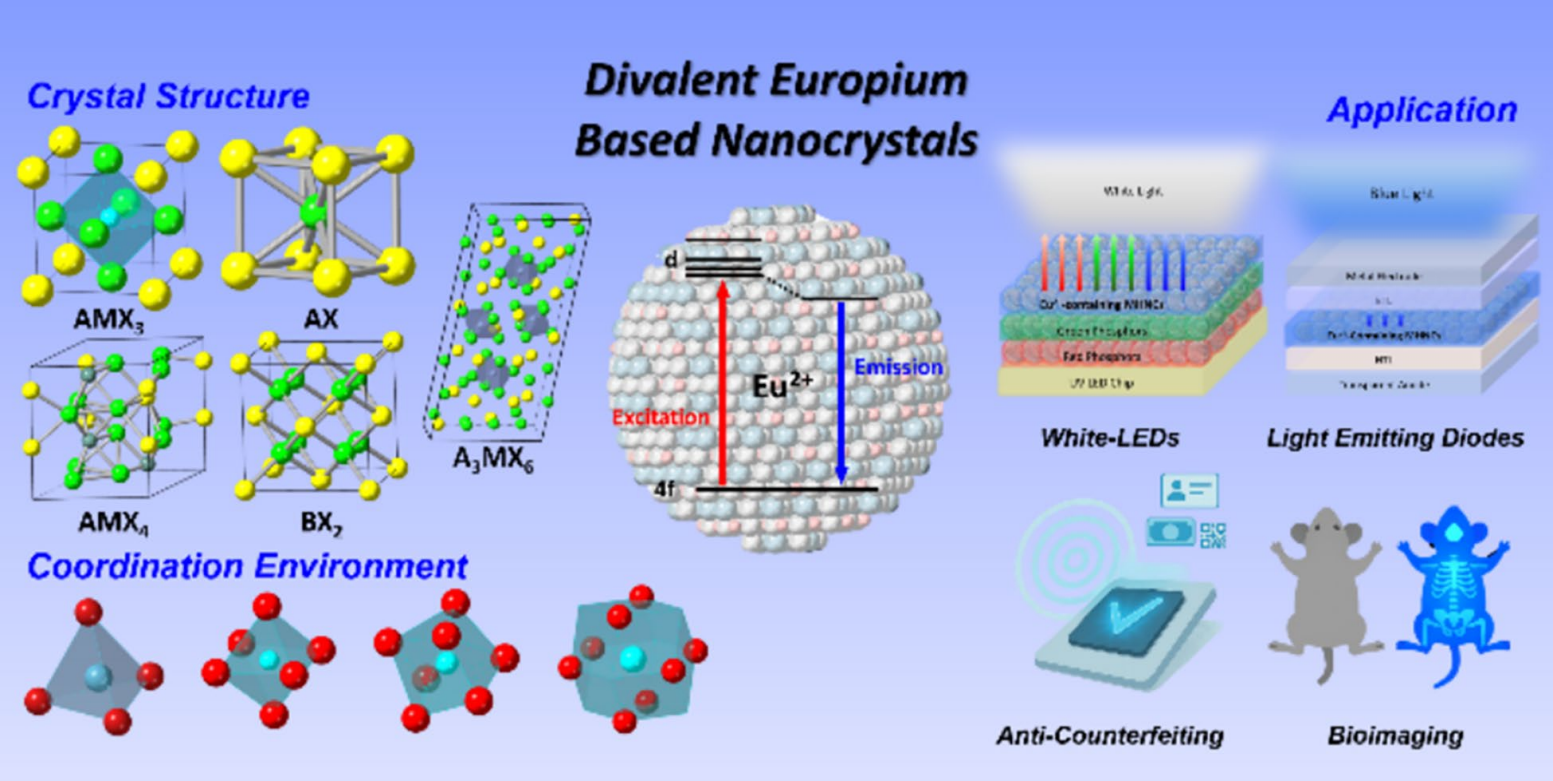

## Introduction

Lanthanide elements are being widely used in various fields, such as displays, magnetic applications, and catalysts, owing to their unique electronic, optical, and magnetic properties [1–4]. The lanthanide series includes elements from lanthanum (atomic number 57) to lutetium (atomic number 71), the ions of which commonly exist as trivalent cations (Ln^3+^) with an electron configuration of [Xe]4f^*n*^ (*n* = 0–14) [5–7]. The partially filled 4f orbitals contribute to the magnetic behaviors of Ln^3+^ ions, owing to the unpaired electrons in these orbitals [8]. In addition, electron transitions between 4f orbitals, which function as optically active centers, result in luminescent characteristics [9]. Because the 4f electrons are shielded by the outer 5s^2^ and 5p^6^ orbitals, their energy levels are not significantly affected by external environmental factors or chemical interactions. Consequently, Ln^3+^ ions exhibit narrow atomic absorption and emission spectra, and their 4f energy levels remain largely unaffected by the chemical environments, as depicted by the Dieke diagram [10, 11]. Furthermore, the electronic transitions between 4f orbitals is forbidden according to the Larporte rule, resulting in long excited-state lifetimes, often in the millisecond range [12]. These optical features enable unique phenomena, such as upconversion luminescence and quantum cutting, facilitated by multiphoton absorption and emission [13–15].

Some lanthanide ions can exist as stable divalent cations (Ln^2+^) [16]. Compared with the trivalent ions, Ln^2+^ ions exhibit distinct electronic structures and photophysical properties, including high radiative emission probability, high extinction coefficients, and short excited-state lifetimes, typically in the nanosecond range [17]. Unlike the shielded 4f orbitals, the 5d orbitals in Ln^2+^ ions are sensitive to the surrounding crystal field, resulting in broad excitation and emission spectra owing to the parity-allowed 4f^n–1^5d ↔ 4f^n^ transitions [18–22]. Each Ln^2+^ ion exhibits distinct optical and physical properties. For instance, Eu^2+^ exhibits a high standard reduction potential, suggesting that Eu^3+^ is readily reduced to Eu^2+^ [23]. Consequently, Eu^2+^ attains a more stable divalent state compared with that of other lanthanide ions. Depending on the host lattice, Eu^2+^ can emit light across a broad range, from ultraviolet (UV) to red, with emission color tunable through its local coordination environment in inorganic compounds such as borates, halides, and pyrophosphates [24–30]. In case of Yb^2+^, the energy of the excited states shifts to the blue range, showing green emission [31]. Yb^2+^ also has high energy resolution and excellent light yield as a potential activator for halide scintillators [32]. Tm^2+^ is considered a promising upconversion activator with high efficiency deep green emission due to its metastable excited states in the red and near-infrared region [33]. Lastly, Nd^2+^ absorbs photons in the NIR region and then produce lower energy states than visible light. This process causes electronic interaction like rotational level doubling, making Nd^2+^ an important element in laser devices [34, 35]. Thus, the luminescence characteristics and tunability of Ln^2+^ have rendered them an attractive research subject alongside the well-established Ln^3+^ systems.

Ln^2+^-based luminescent materials are generally classified into coordination complexes and solid-state materials, with the latter offering superior chemical and optical stability, as well as enhanced luminescence performance. In solid-state systems, Ln^2+^ ions can serve as either dopants in crystal structures such as chalcogenides [36, 37], metal halides [38, 39], and perovskites [40, 41], functioning as emissive centers, or as primary components in inorganic crystalline hosts [42–45]. The luminescence properties of Ln^2+^ solid-state materials, including emission and excitation wavelengths, quantum yield, and lifetime, are affected by various factors, such as the crystal structure, interatomic bond distances, coordination number, and lattice defects of the host. These factors can be modulated through synthesis parameters, enabling precise tuning of the material properties [22, 46, 47]. Furthermore, solid-state phosphors exhibit excellent environmental and structural stability, including thermal resistance [48], long-term durability [49], and resistance to moisture and oxygen [50, 51], making them particularly well-suited for practical device integration.

For the preparation of solid state materials, calcination process of the mixture of metal precursors is widely used as a method for synthesizing solid-state phosphors [42, 52, 53]. Calcination typically requires high temperatures above 700 °C, and in some cases, even exceeding 1000 °C, along with relatively long reaction times. Moreover, due to the high-temperature conditions, both the heating and cooling processes are time-consuming. In contrast, colloidal synthesis offers significant advantages, as reactions can proceed at relatively low temperatures and shorter timeframes, enabling the efficient production of phase-pure nanomaterials. This method allows for the fabrication of nanocrystals (NCs) with uniform morphology and narrow size distribution, while also providing facile control over synthetic parameters to tailor diverse luminescence characteristics. Additionally, colloidally synthesized NCs exhibit excellent dispersibility in solvents, which facilitates their integration into thin films and optical devices with small form factors.

Recently, the development of Ln^2+^-based luminescent inorganic NCs using metal halide nanocrystal (MHNC) hosts has garnered significant research interest [54, 55]. Among the various Ln^2+^ ions, Eu^2+^ has emerged as particularly attractive owing to its favorable luminescence properties and versatile applications, such as white light-emitting diodes (WLEDs) [56, 57], nanothermometers [58, 59], and scintillators [60, 61]. Eu^2+^ serves as a functional component in these applications, owing to its unique luminescent properties. In WLEDs, Eu^2+^ acts as an activator in inorganic phosphors, partially occupying cation sites within the host lattice and functioning as a luminescent center through electronic transitions. Regarding nanothermometers, the luminescence of Eu^2+^ is highly sensitive to temperature changes, whereas Eu^3+^ remains stable under varying conditions. This contrast allows Eu^2+^/Eu^3+^ co-doped inorganic NCs to operate as ratiometric nanothermometers, where Eu^3+^ provides a reliable reference and Eu^2+^ acts as a temperature-responsive probe. Furthermore, Eu^2+^ can serve as an active center in scintillators, efficiently converting excitation energy generated by radiation into visible light, owing to its fast 4f-5d transitions. In addition, there is a growing interest of Eu^2+^-based luminescent NCs for blue emitters in light-emitting diodes (LEDs) owing to deep-blue emission profiles and the narrow PL bandwidth [62, 63]. Ce^3+^-based phosphors and InP quantum dots have been explored as blue emitters for LED applications [64–69]; however, their broad emission bandwidths generally lead to poor color purity. Although certain InP quantum dots exhibit high quantum yields, they typically require additional processing steps to form core–shell structures for enhancing their stability and emission efficiency. In contrast, Eu^2+^-based MHNCs demonstrate highly competitive photoluminescence (PL) characteristics, particularly in terms of narrow-band deep-blue emission and high luminescence efficiency. These advantages render these materials highly suitable for applications requiring precise spectral control. Despite its potential, comprehensive studies on the luminescence properties of Eu^2+^ and its MHNCs remain limited. This review discusses the theoretical aspects of Eu^2+^ luminescence and highlights recent progress in the synthesis of Eu^2+^-based MHNCs. First, we summarize the phenomenological theory from Dorenbos’ perspective and introduce physical approaches for tuning Eu^2+^ emission in solid-state phosphors. Next, we elaborate upon colloidal synthesis methods, which enable precise fabrication of Eu^2+^-based MHNCs under controlled conditions at the nanometer scale. We also address the physical and chemical properties depending on whether Eu^2^⁺ functions as a host or dopant. Overall, this review highlights selected studies on Eu^2^⁺-based MHNCs, focusing on their colloidal synthesis, luminescence properties, and potential applications, thereby suggesting various directions for future research on novel emitters.

## Fundamental understanding of Eu^2+^ luminescence in solid-state materials

**Fig. 1:**
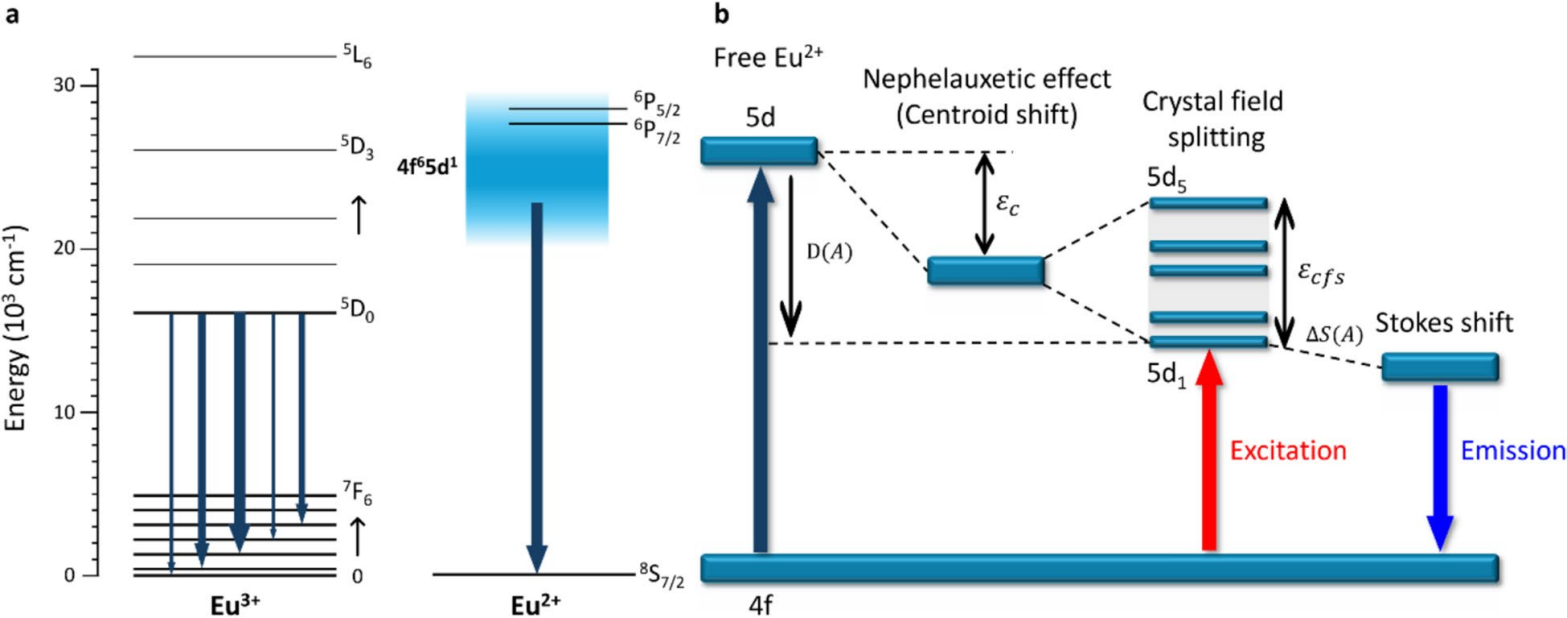
**a** Schematic energy level diagram of Eu^3+^ and Eu^2+^ phosphors. **b** Schematic illustrating the influence of the crystalline environment on the 5d energy levels of Eu^2+^ doped into a host compound.

While Eu^3+^ shows sharp line emission in the red to NIR region, Eu^2+^ exhibits broadband emission across the visible range, particularly in blue region (Fig. 1a). This band emission arises from the parity-allowed 4f^6^5d^1^ → 4f^7^ transition and can be modified by altering the local environment surrounding Eu^2+^. In detail, a free Eu^2+^ ion has a 4.216 eV energy gap between 4f state and 5d state [70]. By incorporating this free Eu^2+^ into the host lattice (A), the energy gap between 4f-5d levels is varied depending on the centroid shift (${\varepsilon }_{c}$), which describes the downward shift in the average energy of the 5d levels, and crystal field splitting (${\varepsilon }_{cfs}$), which denotes the energy separation of the individual 5d orbitals induced by the crystal field [71]. These factors together represent the spectroscopic red shift, D $(A)$ [72]. Upon excitation, the lattice relaxation at the 5d energy levels induces a Stokes shift ($\Delta S(A)$), which is influenced by the type of host [73]. The lowest 5d energy level and $\Delta S(A)$ determine the PL emission wavelength. Therefore, tuning of the host system enables the modulation of both D $(A)$ and $\Delta S(A)$ (Fig. 1b) [74].

${\varepsilon }_{c}$ is related to the expansion of the charge cloud observed in the 5d energy levels, known as the nephelauxetic effect [72, 75]. The weak covalent bond between the 5d orbitals of Eu^2+^ and p orbitals of the surrounding anion expands the 5d orbitals, thereby reducing the Coulomb repulsion between the Eu^2+^ electrons. This enhanced covalent bonding increases ${\varepsilon }_{c}$, leading to a red shift in the emission spectrum. Dorenbos experimentally demonstrated that the nephelauxetic effect can be represented in terms of the anion spectroscopic polarizability (${\alpha }_{sp}$) [76] as

1 $$\begin{array}{c}{\alpha }_{sp}= {\alpha }_{0}\left(X\right)+\frac{b\left(X\right)}{{\chi }_{av}^{2}}\end{array}$$

where ${\alpha }_{0}\left(X\right)$ is the limiting polarizability of anion $X$, $b\left(X\right)$ is the susceptibility of $X$, and ${\chi }_{av}$ is the electronegativity [76]. Thus, ${\varepsilon }_{c}$ is affected by the polarizability of the ligand and can be modulated through anion substitution or by altering the number of covalent bonds. In complexes with common metal ions, such changes are correlated with the magnitude of the nephelauxetic effect of the ligand, which typically follows the order shown below [77, 78].

2 $$\begin{array}{c}{F}^{-}< {H}_{2}O< {NH}_{3}<en<{\left[ox\right]}^{2-}<{\left[NCS\right]}^{-}<{Cl}^{-}<{\left[CN\right]}^{-}<{Br}^{-}< {I}^{-}\end{array}$$

The PL excitation (PLE) and emission spectra are influenced by ${\varepsilon }_{cfs}$. The degree of ${\varepsilon }_{cfs}$ depends on the bond length between the activator ion and coordinating ligand, degree of molecular overlap or covalent bonding, coordination environment, and local symmetry [79]. According to the point-charge model, these effects can be quantified as follows:

3 $$\begin{array}{c}{D}_{q}=\frac{Z{e}^{2}{r}^{4}}{6{R}^{5}}\end{array}$$

where ${D}_{q}$ is the magnitude of the 5d energy-level splitting, $Z$ denotes the charge of the anion, $e$ is the electronic charge, $r$ is the radius of the d-orbital wave function, and $R$ is the bond length [77]. ${\varepsilon }_{cfs}$ is inversely proportional to $R$, indicating that D $(A)$ increases as the bond length decreases. [80, 81]. Furthermore, a more distorted polyhedron has been noted to be correspond to a larger ${\varepsilon }_{cfs}$[82]. Therefore, the red shift can be modulated by adjusting the bond length or coordination number for the host polyhedron. $\Delta S(A)$ represents the energy gap between the maxima of the excitation and emission bands. Upon excitation, electrons move from the 4f ground state to the 5d excited state and then return to the 4f ground state. However, the actual emission energy is lower than the excitation energy owing to the energy loss caused by relaxation. As greater structural rigidity of the host tends to reduce $\Delta S(A)$, the magnitude of $\Delta S(A)$ can be selectively controlled [74, 82].

In summary, the luminescence properties of Eu^2+^ in solid-state systems are highly sensitive to external factors, such as the type and structure of the host lattice, symmetry of the activator site, and coordination environment [83–85]. These factors influence the energy levels of the Eu^2+^ 5d orbitals, enabling control over the emission wavelengths. For instance, in host lattices with lower coordination numbers, the splitting of the 5d orbitals increases, lowering the energy of the lowest 5d level and producing a red-shifted emission [86]. In addition, the coordination environment plays a crucial role, as coordination interactions can modulate the energy levels of the 5d orbitals. Increased covalency between the Eu^2+^ ion and anionic ligands within the host lattice further enhances this effect, resulting in a red-shifted emission wavelength [87]. Therefore, the local environment surrounding Eu^2+^ must be precisely controlled for achieving efficient blue emission in solid-state phosphors.

## Colloidal synthesis of MHNCs

In the colloidal synthesis of MHNCs, hot-injection and heating-up techniques are widely used. Although these approaches follow different synthetic routes, both are well known for producing highly uniform and monodisperse NCs [88]. In the hot-injection method, highly reactive precursors are rapidly injected into a hot reaction solution containing surfactants [89]. This rapid injection leads to immediate supersaturation, which induces burst nucleation over a short period. As the precursor concentration decreases, nucleation ceases and only particle growth continues. By separating the nucleation and growth stages, the hot-injection method offers excellent control over particle size distribution, yielding highly monodisperse NCs. This method is primarily used for synthesizing binary or ternary MHNCs [90–94]. Typically, metal halide precursors are dissolved in high-boiling-point organic solvents such as oleylamine, octadecene, and oleic acid. Prior to the reaction, a degassing step is performed to eliminate moisture and oxygen, which could adversely affect NC formation. The precursor solution for injection is separately prepared as a stock solution. The main reaction vessel is purged with N_2_ or Ar to establish an inert atmosphere. The stock solution is swiftly injected into the reaction mixture at elevated temperatures of 250–350 °C, initiating NC formation. Once the reaction is complete, the solution is cooled to room temperature (25–40 °C) and purified to obtain the desired NCs.

In contrast, the heating-up method involves mixing all precursors, ligands, surfactants, and solvents at low temperatures, followed by gradually increasing the temperature to induce NC formation [95]. Unlike the hot-injection method, the heating-up method often involves overlapping nucleation and growth stages. As the temperature rises, the precursors decompose progressively, resulting in sustained nucleation over an extended period. This approach is simple and scalable, as it is involves a one-pot reaction without requiring additional injection steps. The heating-up method is widely used in the synthesis of fluoride-based NCs, such as AMF_4_ [96–98], MF_3_ [99–101], and AeF_2_ [102, 103], where A represents a monovalent alkali metal, M refers to a trivalent metal cation, and Ae denotes a divalent alkaline earth metal. A typical synthesis process involves dissolving metal acetates, metal trifluoroacetates, or metal halides in high-boiling-point organic solvents, followed by degassing and reaction under an inert atmosphere at a controlled temperature and time. As the temperature increases, NCs are gradually formed. Upon completion of the reaction, the solution is cooled and purified to isolate the target NCs. This approach is extensively applied for synthesizing fluoride-based NCs exhibiting upconversion luminescence, enabling the conversion of infrared light into visible emission. Moreover, this method can also be adapted for the synthesis of binary or ternary MHNCs [104, 105]. Both the hot-injection and heating-up methods enable precise control over NC morphology and size by tuning parameters such as reaction temperature, reaction time, precursor type, and concentration. By precisely controlling these synthetic conditions, the structural and optical properties of NCs can be modulated.

## Luminescent NCs with Eu^2+^ as the host elements

Depending on how Eu^2+^ is incorporated into the host lattice, it may function either as a dopant ion—substituting into an existing crystal lattice—or as a framework cation forming the host matrix. The structural role of Eu^2+^ fundamentally influences the synthesis conditions, local coordination environment, and resulting photoluminescence behavior. When introduced as a dopant, Eu^2+^ acts as a luminescent center in dielectric hosts. In certain cases, the host may absorb photons and transfer energy to Eu^2+^, inducing Eu^2+^-based luminescence. In contrast, when acting as the host cation, Eu^2+^ forms the primary framework of luminescent materials. In this scenario, Eu can serve both as the structural cation in the host matrix and as an active luminescent center. Depending on the composition, various crystal structures may be formed. The reported compositions in ternary systems include AEuX_3_, A_3_EuX_6_, and AEu_2_X_5_, whereas EuX_2_ is a representative structure in binary systems. Here, A denotes an alkali metal, and X represents a halogen element. Among these compositions, AEuX_3_ has attracted significant attention as an environmentally friendly, blue-emitting perovskite, offering a high PLQY with a narrow emission bandwidth. In this structure, Eu^2+^ occupies divalent cation sites in an octahedral coordination environment. The ionic radius of Eu^2+^ (117 pm) is comparable to that of Pb^2+^ (119 pm) [106], satisfying the Goldschmidt tolerance factor required for forming a stable perovskite structure [107]. Consequently, AEuX_3_ is considered a promising candidate for lead-free, eco-friendly perovskites. Notably, AEuX_3_ exhibits excellent optoelectronic properties, including high PL efficiency, outstanding color purity, and high carrier mobility. For instance, CsEuBr_3_ demonstrates a high exciton binding energy and radiative recombination rate, regardless of size confinement [42]. These features are attributable to the spatial confinement of Eu 4f and 5d orbitals and the strong overlap of electron and hole charge densities within the [EuBr_6_]^4−^ octahedra. These characteristics highlight the potential of AEuX_3_ as a narrow-band blue emitter.

Among MHNCs, perovskite NCs have been widely explored [108–111]. Perovskites typically possess the formula ABX_3_ and are composed of three-dimensionally interconnected octahedral structures. The physical properties of perovskites can be finely tuned by varying the combination of A-site cations and X-site halide anions. Pb-based perovskites have been extensively studied owing to their high PL efficiency and narrow full-width at half-maximum (FWHM). Alam et al. first reported the synthesis of CsEuBr_3_ perosvkite NCs [112]. Specifically, CsEuBr_3_ NCs were synthesized using the hot-injection method, originally proposed by Protesescu et al. for developing CsPbX_3_ NCs [90]. The obtained CsEuBr_3_ NCs exhibited a spherical shape with a diameter of 43 ± 7 nm. X-ray diffraction (XRD) analysis revealed that the main diffraction peaks of CsEuBr_3_ NCs were distinct compared with those of CsBr and EuBr_2_, confirming the formation of a unique CsEuBr_3_ crystal structure. The intense blue emission at 413 nm was attributable to the 4f^7^ → 4f^6^5d^1^ transition of Eu^2+^ within the CsEuBr_3_ crystal lattice. The energy of the 5d orbitals was perturbed by the crystal structure, leading to unique optical emission characteristics. Time-resolved photoluminescence (TRPL) measurements showed an average lifetime of 262.6 ns, reflecting fast electron transitions. Additionally, the CsEuBr_3_ NCs exhibited a high PL quantum yield (PLQY) of 39%, indicating excellent optical performance. This research highlighted the potential of CsEuBr_3_ as an environmentally friendly blue-emitting material for optoelectronic applications.

**Fig. 2:**
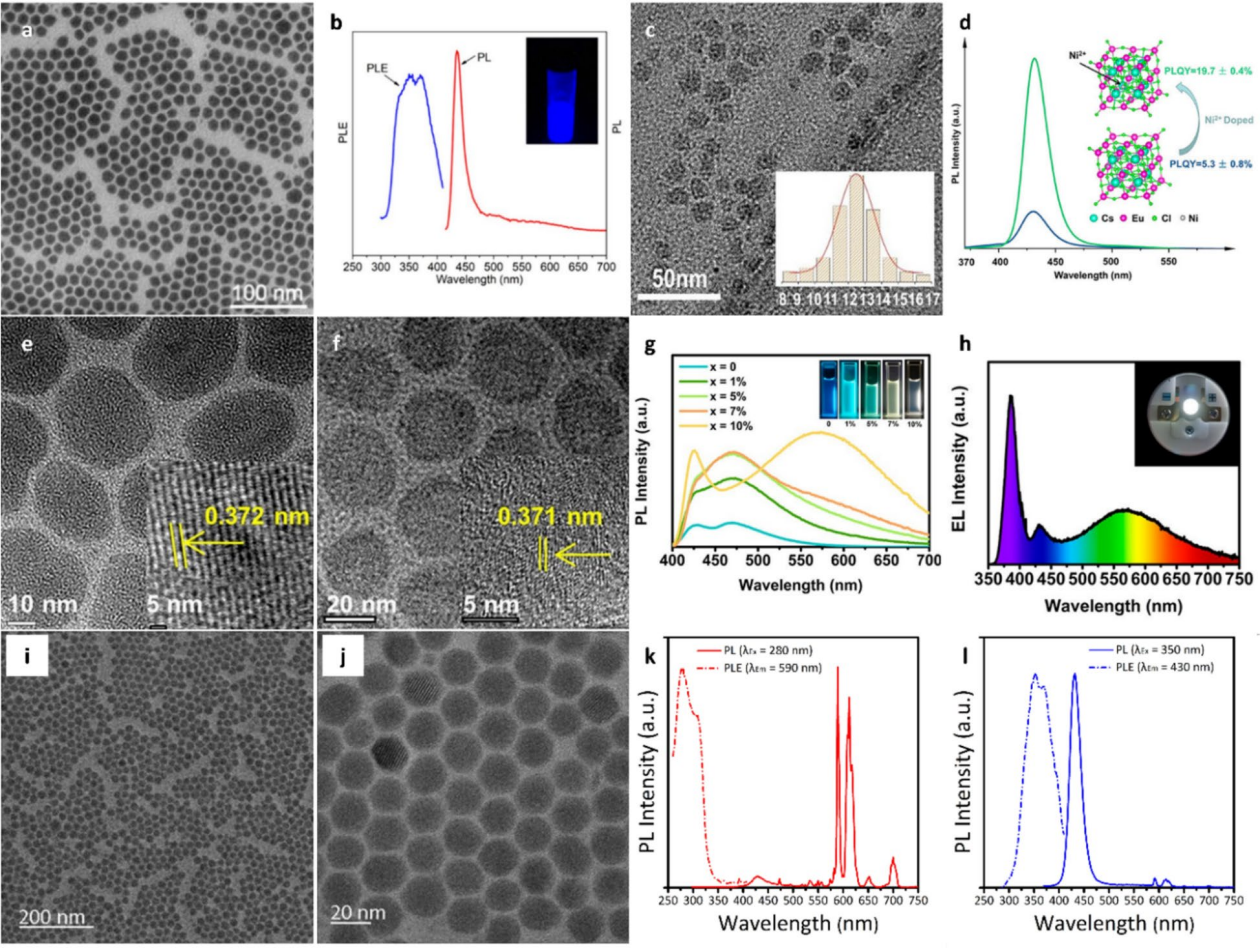
**a** Transmitted electron microscope (TEM) image, **b** photoluminescence (PL) emission and PL excitation (PLE) spectra of CsEuCl_3_ nanocrystals (NCs). Reproduced with permission from [114]. Copyright 2020, American Chemical Society. **c** TEM image of 7.3% Ni^2+^-doped CsEuCl_3_ perovskite NCs (PNCs) and corresponding size distribution (inset). **d** Comparison of PL spectra and PL quantum yield (PLQY) values between CsEuCl_3_ NCs and 7.3% Ni^2+^-doped CsEuCl_3_ NCs [115]. Copyright 2023, American Chemical Society. TEM images of **e** RbEu_2_Cl_5_ and **f** RbEu_2_Cl_5_:10% Ce^3+^ NCs. **g** PL spectra of RbEu_2_Cl_5_:Ce^3+^ NCs with varying Ce^3+^ doping concentrations. **h** Electroluminescence (EL) spectrum of RbEu_2_Cl_5_:10% Ce^3+^ NC-based WLED; inset shows the photograph of the working WLED. Reproduced with permission from [116]. Copyright 2023, American Chemical Society. **i** Low-magnification and **j** high magnification TEM images of Cs_3_EuCl_6_ NCs. **k** PL spectra of Cs_3_EuCl_6_ NCs excited at 280 nm (red line) and PLE spectra monitored at 590 nm (red dashed line). **l** PL spectra of Cs_3_EuCl_6_ NCs excited at 350 nm (blue line) and PLE spectra monitored at 430 nm (blue dashed line). Reproduced with permission from [117]. Copyright 2023, Royal Society of Chemistry.

Notably, the synthesis of phase-pure Cs–Eu halide perovskites remains challenging owing to the oxidation sensitivity of Eu^2+^ ions, which renders them highly unstable [113]. Huang et al. synthesized phase-pure CsEuCl_3_ by reducing Eu^3+^ to Eu^2+^ [114]. The synthesized NCs exhibited a crystal structure of CsEuCl_3_ (*p4mm*, tetragonal) and an average size of 15 nm (Fig. 2a). The CsEuCl_3_ NCs exhibited a sharp blue emission centered at 435 nm with a narrow FWHM of 19 nm (Fig. 2b). The PLQY was measured to be 2 ± 0.3%, which increased to 5.7 ± 0.3% after surface treatment with 1-butyl-1-methylpyridinium chloride. This is because the ionic ligands acted as a chlorine source, replacing the chloride vacancies on the NC surface. The CsEuCl_3_ NCs were stable in toluene for several months. However, when drop-cast onto a glass substrate and exposed to air, they decomposed and oxidized owing to the presence of moisture and oxygen, resulting in red emission associated with Eu^3+^ transitions. To prevent this degradation, the NCs were encapsulated in a polymethyl methacrylate polymer matrix. The encapsulated NCs exhibited long-term optical stability while maintaining blue emission centered at 436 nm. Even after continuous laser irradiation (380 min), the PL peak position and intensity remained nearly unchanged.

Using this modified synthesis method, Li et al. prepared CsEuBr_3_ NCs through the hot injection of trimethyl bromosilane (TMS-Br) into a Eu^3+^ precursor solution [118]. CsEuBr_3_ NCs were formed through reaction crystallization, depending on the ratio of Eu to Cs, injection temperature, and reaction time. Notably, the reaction time has the most significant effect on the PLQY of CsEuBr_3_ NCs owing to its influence on the defect concentration. To quantify this impact, the Urbach tail was analyzed, revealing that a larger Urbach energy (*E*_U_) corresponded to a higher defect density. The reaction time of 45 min resulted in the smallest *E*_U_, indicating minimal defects. The obtained CsEuBr_3_ NCs exhibited a spherical morphology with a diameter of 15.90 ± 0.33 nm. In the PL spectrum, these NCs displayed an emission peak at 443 nm, with a narrow FWHM of 28.5 nm, showing high-purity deep-blue emission with a PLQY of 93.51%. This high PLQY was attributable to the passivating effect of oleylamine ligands on halide defects and the high crystallinity of the NCs. Complete reduction of Eu^3+^ to Eu^2+^ was confirmed by the absence of the characteristic red emission of Eu^3+^ ions, verifying that Eu^2+^ was responsible for the blue emission. First-principles calculations and PL measurements further confirmed that the high luminescence efficiency and stability of Eu^2+^ were attributable to transitions from Eu-5d to Eu-4f/Br-4p. Furthermore, the unique [EuBr_6_]^4−^ octahedral structure, characterized by overlap of electron and hole densities, further increased the radiative recombination rates, contributing to fast radiative recombination and efficient emission [42]. With increasing temperature, the PL intensity decreased, and the blue emission slightly shifted toward cyan. This behavior was attributable to lattice expansion, which slightly reduced the potential energy of the Eu^2+^ 5d orbital, contributing to temperature-dependent PL changes while maintaining emission efficiency. These CsEuBr_3_ NCs were embedded in polydimethylsiloxane to create flexible blue light-emitting films. These films displayed uniform blue emission under UV light and stability against water and heat. These composite films are promising for use in LCD backlighting, offering excellent color reproduction and a wide color gamut for high-efficiency display applications.

The phase, size, and composition of NCs vary significantly depending on the type of ligand used [119]. Ha et al. revealed how the amount and type of ligand influence phase evolution [107]. The authors used two Br precursors: oleylammonium bromide (OLAMHBr) and trioctylphosphine dibromide (TOPBr_2_). The obtained CsEuBr_3_ NCs displayed different optical and structural properties. Among them, the NCs synthesized using TOPBr_2_ exhibited superior optical properties, forming a pure CsEuBr_3_ perovskite phase (orthorhombic, space group *Pbnm*) with a PLQY of 40.5% and narrow FWHM of 24 nm. Compared with OLAMHBr-based NCs, the emission peak was red-shifted by approximately 10 nm, and the PLQY was significantly enhanced. This red shift was attributable to variations in the stoichiometries of CsEuBr_3_ and resulting distortions in the octahedral coordination of Eu^2+^. The TOPBr_2_-based NCs predominantly consisted of well-crystallized CsEuBr_3_, exhibiting enhanced PL owing to the stable formation of [EuBr_6_]^4−^ octahedral units. In contrast, the OLAMHBr-based NCs contained significant amounts of CsBr and other byproducts. The lower PLQY in the OLAMHBr-based NCs was linked to the incorporation of excess Eu^2+^ into the CsBr lattice, leading to structural distortions. Overall, the phase evolution was influenced by the precursor stoichiometries, emphasizing the critical role of precursor choice in determining the structural and optical properties of CsEuBr_3_ NCs.

The luminescence properties of materials can be significantly enhanced by co-doping with additional ions. The incorporation of a secondary ion alongside the primary dopant has been noted to facilitate the optimization of energy-transfer mechanisms, enabling precise tuning of emission wavelengths [120, 121]. For example, Zhang et al. synthesized Ni^2+^-doped CsEuCl_3_ NCs (cubic, *pm-3 m*) and suggested that Ni^2+^ doping enhanced the PLQY of undoped CsEuCl_3_ NCs [115]. The Ni^2+^-doped CsEuCl_3_ NCs have a polyhedral contour with an average particle size of 12 nm (Fig. 2c). Both pure CsEuCl_3_ NCs and Ni^2+^-doped CsEuCl_3_ NCs exhibited bright blue-violet emission centered at 430.6 ± 0.6 nm. The PLQY varied with the Ni^2+^ doping concentration, reaching a maximum value of 19.7% at a doping level of 7.3% (Fig. 2d). Moreover, the average PL lifetime increased from 2.71 ns to 3.27 ns, attributable to a high radiative recombination rate (*k*_r_) of 60.2 μs^–1^ and a low nonradiative recombination rate (*k*_nr_) of 245.6 μs^–1^. The 7.3% Ni^2+^-doped NC film maintained more than 80% of its PL intensity over 15 min, showing significantly improved stability compared with pure NCs. Ni^2+^ doping reduced structural distortion by bringing the tolerance coefficient of the perovskite closer to 1. This suggests that B-site doping is an effective approach to improve the performance of CsEuCl_3_ NCs.

Co-doping in MHNCs induces lattice distortion and enhances electron–phonon coupling, thereby improving the luminous efficiency of self-trapped excitons (STEs). Fu et al. synthesized quasi-two-dimensional RbEu_2_Cl_5_ NCs through a hot-injection method [116]. Ce^3+^ ions with varying concentrations were incorporated into RbEu_2_Cl_5_ to induce lattice distortions and enhance PL performance. During synthesis, oleylamine acted as a reducing agent to convert Eu^3+^ to Eu^2+^ and maintained a reducing atmosphere to prevent Eu^2+^ oxidation. Both RbEu_2_Cl_5_ NCs and RbEu_2_Cl_5_:Ce^3+^ NCs exhibited a highly crystalline monoclinic perovskite-type structure (space group *P21/C*) with sizes below 30 nm (Figs. 2e and f). The RbEu_2_Cl_5_ NCs exhibited two sharp blue emissions at 430 and 470 nm, attributable to the ^4^f_6_^5^d_1_ → ^4^f_7_ electronic dipole transition of Eu^2+^ in different coordination environments (coordination numbers 8 and 7). Additionally, a broad shoulder emission was observed at 510 nm, originating from STEs (Fig. 2g). Doping with Ce^3+^ ions induced crystal lattice distortion in RbEu_2_Cl_5_. This distortion led to a red shift in the emission band and also strengthened electron–phonon coupling, thereby enhancing the PL intensity of STE emission. Consequently, the PL intensity of all peaks increased upon Ce^3+^ doping. The 430 and 470 nm peaks became more intense, while the 510 nm peak underwent a red shift. Furthermore, the PLQY of RbEu_2_Cl_5_:Ce^3+^ NCs (5.51%) was higher than that of RbEu_2_Cl_5_ NCs (3.39%). These results confirm that Ce^3+^ doping enhanced the electron–phonon coupling in RbEu_2_Cl_5_:Ce^3+^ NCs, resulting in stronger STE emission and self-activated white-light emission. Owing to their favorable white luminescence, RbEu_2_Cl_5_:Ce^3+^ NCs represented promising single components for lead-free, self-activated WLEDs (Fig. 2h). WLEDs fabricated using these NCs achieved a color rendering index of 70 and a correlated color temperature (CCT) of 4793 K. This study introduces a new lead-free europium halide perovskite for potential self-activated WLED applications.

In zero-dimensional (0D) structures, excitons are strongly localized within individual NCs, resulting in high internal quantum efficiency and narrow emission linewidths [122]. The optical properties of 0D metal halides can be tuned by tailoring their composition, and various 0D metal halide structures, such as Cs_4_PbBr_6_, Cs_3_Cu_2_I_5_, and Cs_3_LnCl_6_, have been reported [123–125]. Lee et al. synthesized 0D Cs_3_EuCl_6_ NCs with a monoclinic structure and a size of 19.51 ± 1.72 nm (Figs. 2i and j) [117]. The 0D Cs_3_EuCl_6_ exhibits multicolor PL properties, which can be dynamically tuned by varying the excitation wavelength. Under 280 nm excitation, characteristic red PL originating from the f–f transitions of Eu^3+^ ions was observed (Fig. 2k). More importantly, blue PL was observed, with emission centered at 430 nm under an excitation wavelength of 350 nm, attributable to Eu^2+^ ions (Fig. 2l). The PLQY of blue PL was measured to be 5.38%, and low-temperature PL measurements showed that the intensity of blue PL increased with decreasing temperature, owing to reduced thermal quenching and increased radiative recombination. Under UV illumination, these Cs_3_EuCl_6_ NCs displayed vivid color changes depending on the excitation wavelength, due to the selective excitation of either Eu^3^⁺ or Eu^2^⁺ emission. Tunable luminescence properties could be leveraged to conceal security patterns or marks and reveal them only under UV irradiation. In addition, the security marks could be transferred to various substrate using printing technology based on the NC solution, enhancing anti-counterfeiting functionality.

**Fig. 3:**
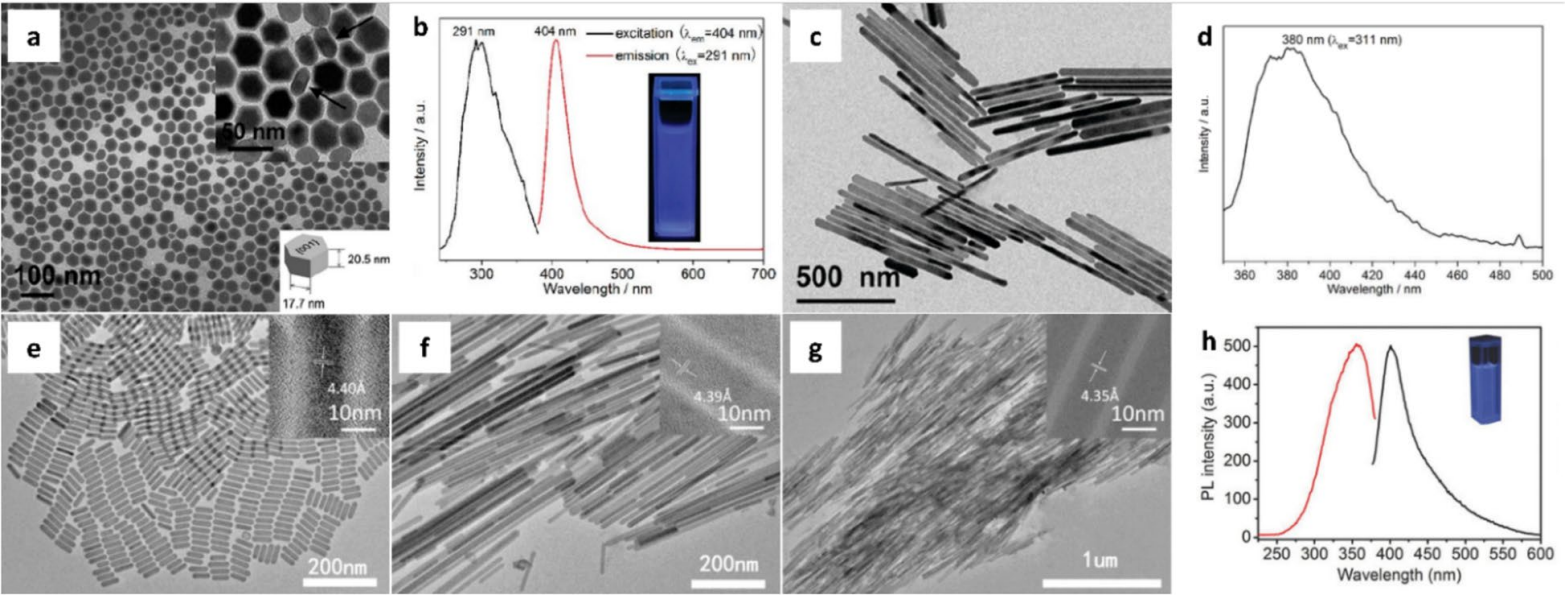
**a** TEM images, **b** PLE (black line) and PL (red line) spectra of EuCl_2_ nanoprisms. **c** TEM image, **d** PL spectrum of EuCl_2_ nanorods. Reproduced with permission from [126]. Copyright 2011, American Chemical Society. TEM images of EuCl_2_ NCs with different urea concentrations: **e** 0.3 mmol, **f** 0.1 mmol, and **g** 0 mmol. **h** PLE (red line) and PL (black line) spectra of EuCl_2_ NCs. Reproduced with permission from [127]. Copyright 2018, Wiley.

Despite many studies focusing on ternary Eu^2+^-based MHNCs including perovskites, the number of reports on binary Eu^2+^-based MHNCs remains limited. Jiang et al. reported the optical properties of binary metal halides by controlling their morphology through the selection of different precursor materials, highlighting that the optical properties are significantly influenced by the morphology of NCs [126]. Eu(CCl_3_COO)_3_·2H_2_O was used as the Eu source for the synthesis of EuCl_2_ nanoprisms, whereas Eu(CH_3_COO)_3_·H_2_O was used for the synthesis of EuCl_2_ nanorods. These precursors were reduced from Eu^3+^ to Eu^2+^ in OAm solvent and in this process, acetamidine hydrochloride and picolinamidine hydrochloride were thermally decomposed at 300 ℃ to produce ammonia. It is possible to successfully achieve the reduction of Eu under ambient conditions rather than an inert gas environment. The synthesized EuCl_2_ exhibited an orthorhombic structure with a space group of *Pbnm*. The nanoprisms have a length of 17.7 ± 1.7 nm and a thickness of 20.5 ± 1.6 nm (Fig. 3a). Under 291 nm excitation, a bright and broad blue emission centered at 404 nm was observed, which is attributed to the Eu^2+^ 4f^6^5d^1^ to 4f^7^ transition (Fig. 3b). When the EuCl_2_ nanoprisms were stored in air for 6 months to examine their stability, the band at 404 nm remained very stable, and the characteristic emission of Eu^3+^ was observed at 613 nm. It suggests EuCl_2_ nanoprisms have high air stability unlike the previous binary Eu^2+^-based compounds. Meanwhile, the nanorods have a length of 180–800 nm and a diameter of 34.4 ± 7.5 nm (Fig. 3c). They exhibited an emission band centered at 380 nm, which is blue-shifted by 24 nm compared with the 4f-5d transition of the nanoprisms (Fig. 3d). The PLQY of the nanoprisms and nanorods were 7.3% and 1.1%, respectively, which was more than 6 times higher in the case of the nanoprisms. This is because the Eu^2+^-Eu^2+^ concentration quenching is efficiently reduced in the case of NCs with smaller particle sizes and uniform surface Eu^2+^ sites. This study provides the insights that the optical performance of EuCl_2_ can vary depending on the shape of the NCs.

In colloidal synthesis, Eu^3+^ is commonly reduced to Eu^2+^ in presence of oleylamine which is used as the ligands as well as reducing agents. In addition, additional reducing agents can be utilized for the reduction of Eu^3+^ to Eu^2+^. Zhao et al. reported a one-pot colloidal heating-up method using urea as a reducing agent [127]. EuCl_2_ NCs were synthesized using EuCl_3_(H_2_O)_6_, urea, oleic acid, oleylamine, and 1-octadecane. Ammonia gas released from urea and oleylamine were used to reduce Eu^3+^ to Eu^2+^. The particle morphology depended on the amount of urea added. When 0.3 mmol of urea was used, uniform rod-shaped particles with a length of 81.3 nm and thickness of 20.2 nm were obtained (Fig. 3e). As the amount of urea decreased, the particle length increased. With 0.1 mmol of urea, wire-shaped particles with a length of 517.8 nm were synthesized (Fig. 3f), while in the absence of urea, fiber-like particles with a length of 1325.8 nm were obtained (Fig. 3g). Therefore, urea functioned as both a reducing and morphology-directing agent. Upon irradiation at 360 nm, a strong blue emission peak appeared at 400 nm (FWHM = 32 nm) (Fig. 3h), corresponding to electron transition from the 4f orbital to 5d orbital. The authors reported that the bright emission was a result of the effective energy transition of oleylamine and oleic acid solvents. The measured PL lifetime was 2.63 ns, and the PLQY at room temperature was 6.5%. As the temperature decreased, the emission intensity increased, owing to reduced ligand vibration or rotation on the nanoparticle surface, which facilitated radiative relaxation of excited electrons. Table 1 summarizes the representative Eu^2+^-based MHNCs in which Eu^2+^ functions as the host matrix, as well as lists their compositions, synthesis methods, and optical properties for comparison.

**Table 1:** Different Eu phosphors and their synthesis methods and properties

Role of Eu^2+^	Materials	Synthesis methods	Shape	Size	Color	λ_em_ (nm)	λ_ex_ (nm)	FWHM (nm)	PLQY (%)	Application	Refs.
Host	CsEuBr_3_	Hot injection	Spherical	43 ± 7 nm	Bright blue	413	320, 355	30	39	N/A	[112]
Host	CsEuCl_3_	Hot injection	N/A	15 nm	Blue	435	350	19	2 ± 0.3	N/A	[114]
Host	CsEuBr_3_	Hot injection	Spherical	15.90 ± 0.33 nm	Deep blue	443	360	28.5	93.51	WLED	[118]
Host	CsEuBr_3_	Hot injection	N/A	N/A	Deep blue	430	360	24.2	40.5	N/A	[107]
Host	CsEuCl_3_:Ni^2+^	Hot injection	Polyhedral	12 nm	Bright blue	430.6 ± 0.6	N/A	23.5 ± 0.3	19.7 ± 0.4	N/A	[115]
Host	RbEu_2_Cl_5_:Ce^3+^	Hot injection	N/A	N/A	Blue	430, 470, 510	315, 365, 430, 470	20, 62, 97	3.39	N/A	[116]
Host	Cs_3_EuCl_6_	Heating-up	N/A	19.51 ± 1.72 nm	Deep blue	430	350	26.11	5.38	Anti-counterfeiting inks	[117]
Host	EuCl_2_	Heating-up	Prisms, rods	Prisms;17.7 ± 1.7 nm (length), 20.5 ± 1.6 nm (thickness), rods;180–800 nm (length), 34.4 ± 7.5 nm (diameter)	Deep blue	291	404	N/A	Prisms; 7.3, rods;1.1	N/A	[126]
Host	EuCl_2_	Heating-up	Rods	81.3 nm (length), 20.2 nm (diameter)	Strong blue	360	400	32	6.5	N/A	[127]
Dopant	CsPbBr_3_:Eu^2+^	Hot injection	Cube	8 nm	Bright blue to green	500–550	365	< 30	55–90	LED	[128]
Dopant	CsPbBr_3_:Eu^2+^	Hot injection	Cube	5–10 nm	Blue	442	406	13	N/A	N/A	[54]
Dopant	CsPbBr_3_:Eu^2+^@SiO_2_	Hot injection	Core–shell	N/A	Blue	465	365	N/A	N/A	In vivo imaging	[129]
Dopant	CsBr:Eu^2+^	Hot injection	N/A	51.5 nm	Bright blue	440	350	31	32.8	WLED	[130]
Dopant	CsCl:Eu^2+^	Hot injection	N/A	10–150 nm	Bright blue	410	325	23.3	4.1	N/A	[55]
Dopant	CsBr:Eu^2+^	Hot injection	Hexagons	40 nm	Deep blue	444	272, 346	30	53.4	WLED	[131]
Dopant	CsBr:Eu^2+^	Hot injection	N/A	38.05 ± 1.24 nm	Deep blue	441	370	30	91.1	LED	[50]

λ_em_ and λ_ex_ denote the emission and excitation wavelengths, respectively, while FWHM refers to the full-width at half-maximum of the emission peak in Eu-based luminescent inorganic nanomaterials

## Luminescent NCs with Eu^2+^ as dopants

As outlined in Table 1, various Eu^2+^-doped MHNCs have been reported; these exhibit distinct blue emission characteristics with narrow FWHM values. Luminescent NCs doped with Eu^2+^ exhibit unique optical properties that are highly dependent on the crystal structure of the host material and the lattice site or coordination environment of the Eu^2+^ ions [132]. Generally, Eu^2+^ replaces specific metal sites in the host NCs or occupies defect sites in the crystal lattice [133, 134]. Successful Eu^2+^ doping typically requires the substitution of Eu^2+^ for a divalent metal cation site where the ionic radius and charge match ensure lattice stability and efficient incorporation. The first 4f^*n*–1^5d (*n* = 1–14) electronic state of Eu^2+^ is positioned below the conduction band minimum only when the ion resides in a divalent or monovalent cation site [72]. This configuration enables radiative transitions from the 4f^n–1^5d excited state to the 4f^n^ ground state, resulting in luminescence. Conversely, if the first 4f^n–1^5d level lies above the conduction band minimum, luminescence may be quenched owing to auto-ionization [135]. Hence, the host NCs maintain their own excitonic luminescence, while simultaneously exhibiting the unique Eu^2+^-induced emission [54].

**Fig. 4:**
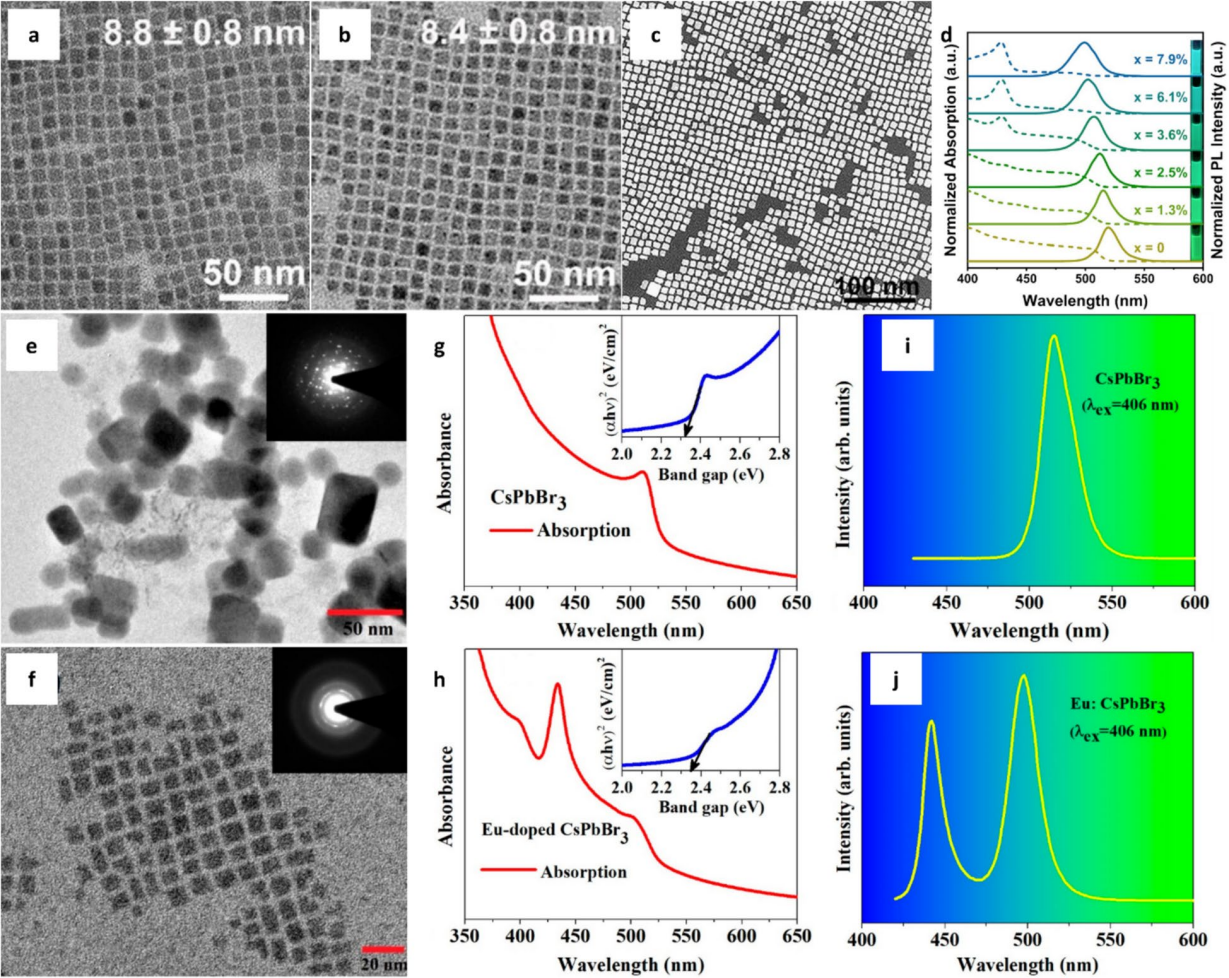
TEM images of **a** pure CsPbBr_3_ and **b** 7.9% Eu^2+^-doped CsPbBr_3_ NCs. **c** STEM image of 7.9% Eu^2+^-doped CsPbBr_3_ NCs. **d** Normalized PL (solid line) and absorption spectra (dashed line) of Eu^2+^-doped CsPbBr_3_ NCs with different Eu^2+^ doping ratios. Reproduced with permission from [128]. Copyright 2022, American Chemical Society. High resolution-TEM images of **e** CsPbBr_3_ and **f** Eu^2+^ doped CsPbBr_3_ NCs. Absorption spectra of **g** CsPbBr_3_ and **h** Eu^2+^ doped CsPbBr_3_ NCs. Emission spectra of **i** CsPbBr_3_ and **j** Eu^2+^ doped CsPbBr_3_ NCs. Reproduced with permission from [54]. Copyright 2023, American Chemical Society

Key host materials for Eu^2+^-doped NCs include halide-based, oxide-based, and sulfide-based NCs. MHNCs, such as CsPbX_3_ and CsX (X = halogen ion), represent a subset of halide-based NCs. Among them, CsPbCl_3_ has been identified as a blue-emitting material; however, its low PLQY limits its practical use. To address this issue, researchers have examined the doping of transition metals such as Cd or Ni into the host lattice [136, 137]. However, the toxicity of heavy metals poses a significant challenge for practical applications. In response to these concerns, increasing research attention has been directed toward blue-emitting phosphors incorporating Eu as a dopant. Eu^2+^ is considered a promising dopant for CsPbX_3_ owing to its similar valence state (2 +) and ionic radius (117 pm) compared with that of Pb^2+^ (119 pm). Given their comparable physical properties, partial substitution or complete replacement of Pb^2+^ with Eu^2+^ can enable the development of high-efficiency, narrow-band blue emitters. CsPbBr_3_, which has a bandgap of approximately 2.3 eV, exhibits high light absorption and quantum efficiencies, emitting green light in the wavelength range of 520–540 nm. Several studies have demonstrated that doping CsPbBr_3_ with Eu^2+^ can modify its bandgap and induce a blue shift in emission. Jin et al. successfully doped Eu^2+^ into the B-site of CsPbBr_3_ NCs, tuning the emission from green to cyan and significantly enhancing both PLQY and stability [128]. Both undoped and doped CsPbBr_3_ NCs with different Eu^2+^ doping ratios exhibited cubelike morphologies with a diameter of approximately 8 nm (Fig. 4a, b). The scanning TEM (STEM) image clearly reveal the uniform cubic morphology of Eu^2+^-doped CsPbBr_3_ NCs, indicating their high monodispersity (Fig. 4c).With increasing Eu^2+^ content in CsPbBr_3_ NCs, both the absorption edge and PL emission peak exhibited a continuous blue shift (Fig. 4d). PLQY initially increased from 55% (undoped) to 90% as the Eu^2+^ concentration increased to 3.6% but declined to 55% with further increase in doping concentration to 7.9%, indicating the existence of an optimal doping range for maximum PLQY. Interestingly, the 7.9% Eu^2+^-doped CsPbBr_3_ NCs displayed enhanced thermal stability, retaining 70% of their initial emission intensity after 5 h of heating. According to first-principles calculations, Eu^2+^ incorporation strengthens Pb–Br bonding, thereby reducing structural distortions and improving stability.

Similarly, Kachhap et al. demonstrated that Eu^2+^ doping in CsPbBr_3_ NCs led to the emergence of blue emission in addition to the characteristic green emission [54]. CsPbBr_3_ NCs were synthesized using the hot-injection method, both with and without Eu^2+^ doping. Both undoped and Eu^2+^-doped CsPbBr_3_ NCs exhibited the orthorhombic phase of CsPbBr_3_ with the space group *Pbnm*. However, owing to size differences, the diffraction peaks of Eu^2+^-doped CsPbBr_3_ NCs were broader than those of the CsPbBr_3_ NCs. The undoped CsPbBr_3_ NCs exhibited a non-uniform structure, deviating slightly from a cubic shape, with an average size of 19 nm (Fig. 4e). In contrast, the Eu^2+^-doped CsPbBr_3_ NCs possessed a uniform cubic shape with an average size of 7 nm (Fig. 4f). UV–Vis absorption and emission spectra clearly demonstrated the optical property differences induced by Eu^2+^ doping. The CsPbBr_3_ NCs exhibited an absorption peak at 510 nm, attributable to the band-to-band transition in CsPbBr_3_ (Fig. 4g). In contrast, the Eu^2+^-doped CsPbBr_3_ NCs presented absorption peaks at 398, 434, and 500 nm (Fig. 4h). The 398 and 434 nm peaks originated from Eu^2+^ 4f-5d transitions, specifically ^7^F_0_ → ^5^L_6_ (398 nm) and ^7^F_1_ → ^5^D_3_ (434 nm). The 500 nm peak corresponded to the band-to-band absorption transition of CsPbBr_3_. In the emission spectra, the undoped CsPbBr_3_ NCs exhibited a sharp green emission centered at 515 nm (FWHM ~ 24 nm), whereas Eu^2+^-doped CsPbBr_3_ NCs displayed two emission peaks at 498 nm and 442 nm, with FWHM values of approximately 20 nm and 13 nm, respectively (Fig. 4i and j). The shift in the green emission of doped NCs resulted from the reduction in average particle size from 19 to 7 nm. The additional emission peak at 442 nm was attributable to the ^4^f_6_(^7^F_j_)^5^d_1_ → ^4^f_7_ transition of Eu^2+^ ions. Furthermore, color coordinate analysis based on the Commission Internationale de l’Éclairage diagram demonstrated the excellent luminous efficacy of the optical radiation and remarkable color purity (> 85%) across various excitation conditions.

These studies are of particular significance as they represent some of the few successful demonstrations of overcoming the intrinsic limitation of CsPbBr_3_ NCs, i.e., their predominant green emission, by inducing additional blue emission through Eu^2+^ doping. The results demonstrate the potential of such materials for blue emission, broadening their applicability in optoelectronic devices. However, the insufficient stability and toxicity of lead halide perovskite NCs limit their widespread use. For instance, in aqueous solutions and biological environments, lead halide perovskite NCs undergo surface erosion, leading to fluorescence quenching and the release of heavy metal ions. These phenomena reduce the luminous intensity, stability, and PLQY of lead halide perovskites. To mitigate these adverse effects, silica-coated lead halide perovskite NCs with enhanced stability and biocompatibility have been developed [129]. Green-emitting CsPbBr_3_:Eu NCs were initially synthesized via the heating-up method, followed by coating with a SiO_2_ shell through the hydrolysis and condensation of a silane precursor. Blue-emitting CsPb(ClBr)_3_:Eu@SiO_2_ NCs were further obtained by tailoring the halogen composition. Both CsPbBr_3_:Eu@SiO_2_ NCs and CsPb(ClBr)_3_:Eu@SiO_2_ NCs showed a core–shell structure, with the core material having an average size of less than 20 nm and the SiO_2_ shell having a thickness of 30 nm. The CsPbBr_3_:Eu@SiO_2_ NCs exhibited green emission at 505 nm, while the CsPb(ClBr)_3_:Eu@SiO_2_ NCs showed blue emission at 465 nm. XRD results confirmed that both CsPbBr_3_:Eu@SiO_2_ NCs and CsPb(ClBr)_3_:Eu@SiO_2_ NCs possessed a perovskite structure without any impurities, consistent with CsPbBr_3_. The surface coating of SiO_2_ improved the luminescence stability in water and inherently reduced Pb^2+^ leakage. Moreover, Eu^2+^ doping mitigated the reduction in PLQY induced by the SiO_2_ coating and further suppressed Pb^2+^ release. Furthermore, silica-coated lead halide perovskite NCs exhibited efficient luminescence in water and high biocompatibility, rendering them suitable for cell imaging.

**Fig. 5:**
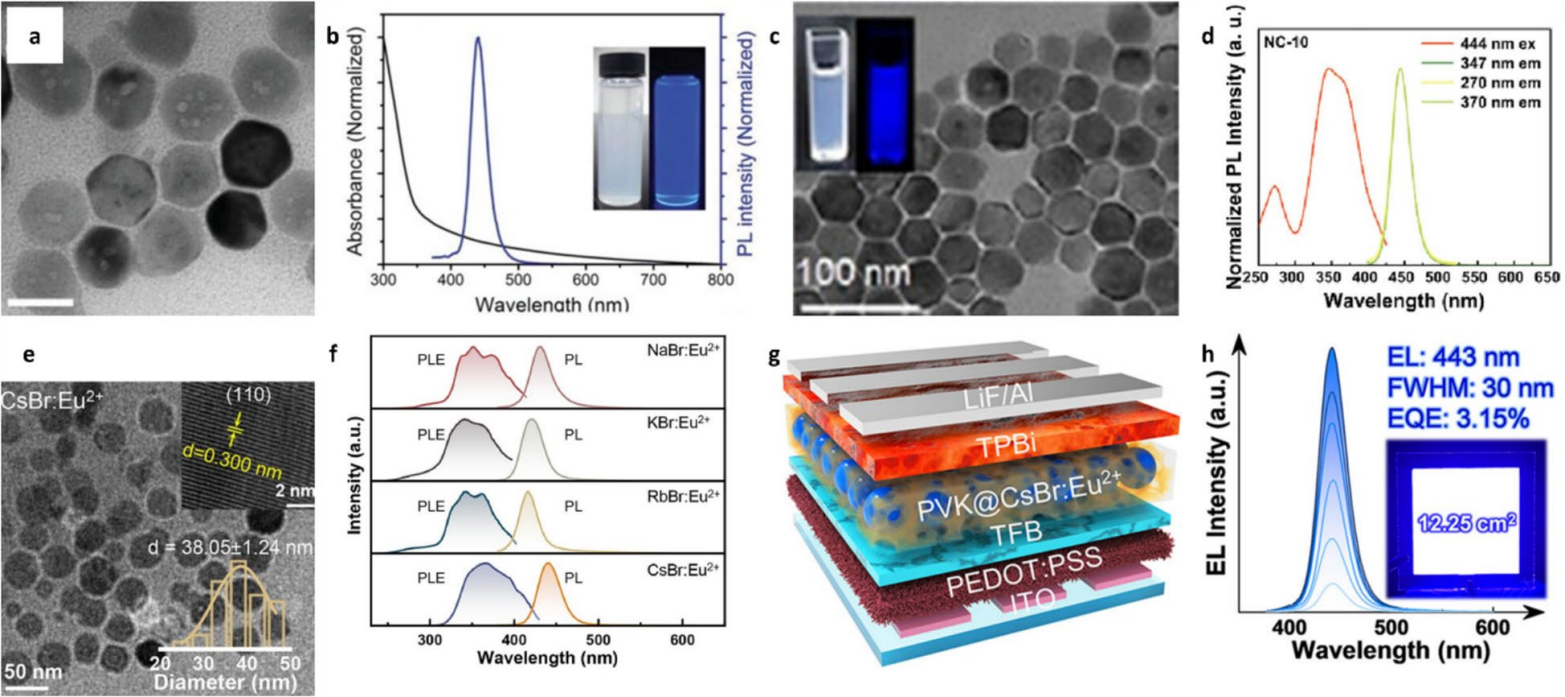
**a** TEM image of CsBr:Eu^2+^ NCs (scale bar: 50 nm). **b** Absorption and PL spectra of CsBr:Eu^2+^ NCs. Reproduced with permission from [130]. Copyright 2019, Wiley. **c** TEM image of CsBr:Eu^2+^ NCs and photographs under daylight (left) and under 365 nm (right). **d** PLE and PL spectra of CsBr:Eu^2+^ NCs. Reproduced with permission from [131]. Copyright 2024, American Chemical Society. **e** TEM image of CsBr:Eu^2+^ NCs. **f** PLE and PL spectra of ABr:Eu^2+^ NCs. **g** Device structure and **h** EL spectra of the deep-blue LEDs based on CsBr:Eu^2+^ NCs. Reproduced with permission from [50]. Copyright 2024, American Chemical Society.

Notably, Eu^2+^ doped is not confined to perovskite structures, and this doping strategy has also been explored in a broader class of halide compounds with the general formula AX (where A is an alkali metal, i.e., Na⁺, K⁺, Rb⁺, Cs⁺; and X is a halide ion, i.e., Cl⁻, Br⁻, I⁻). These alkali halides exhibit diverse optical behaviors and tunable emission characteristics. Yang et al. synthesized Eu^2+^-doped CsBr NCs using the hot-injection method [130]. The average size of the synthesized nanoparticles was 51.5 nm (Fig. 5a). The synthesized CsBr:Eu^2+^ NCs exhibited luminescence at 440 nm under 350-nm excitation with an FWHM of 31 nm (Fig. 5b). Moreover, the CsBr:Eu^2+^ NCs displayed absorption peaks of 272 and 345 nm, corresponding to the splitting of Eu^2+^ 5d orbitals. The PLQY, measured to be 32.8%, remained stable for at least 60 d at room temperature. The decay time of CsBr:Eu^2+^ NCs was 342 ns, attributable to the 4f^6^5d^1^–4f^7^ transitions of Eu^2+^ in Eu^2+^-V_Cs_ isolated dipole centers. Co-doping with Ca^2+^ reduced the nanoparticle size (18.9 nm) without varying the emission wavelength. The size reduction was attributable to Ca^2+^ doping, which increased the nucleation rate owing to the high melting point of Ca, and to Ca^2+^ ions occupying the NC surface, which inhibited further particle growth. CsBr:Eu^2+^ NCs exhibited emission at 440 nm with an FWHM of 29 nm. The narrower FWHM and improved color purity were attributable to the reduced internal stress from Eu^2+^ doping, which stabilized the crystal structure. Furthermore, the CsBr:Eu^2+^ NCs were used to fabricate WLEDs, demonstrating an optical efficiency of 1.74 lm W^–1^ under a 30 mA forward current.

Building upon this work on Eu^2+^-doped AX NCs, Kim et al. prepared Eu^2+^-doped CsCl NCs using the hot-injection method [55]. TEM analysis confirmed that the NCs had a cubic morphology with rounded edges, ranging in size from 10 to 150 nm. Under 325-nm excitation, the CsCl:Eu^2+^ NCs exhibited strong blue emission at 430 nm (FWHM = 23.3 nm) with a PLQY of 1.9%. TRPL analysis revealed a nonradiative recombination time of 4.97 ns, a radiative recombination time of 40.1 ns, and an average lifetime of 6.45 ns. As the temperature increased, the peak intensity decreased, and a slight blue shift in emission energy was observed. This temperature-dependent shift in emission wavelength was attributable to the change in the crystal lattice constant caused by thermal expansion.

To further optimize luminescence and clarify the NC growth mechanism, Qing et al. comprehensively explored the effects of oversaturated Eu^2+^ doping on both structural evolution and photophysical performance [131]. The hot-injection method was used to synthesize CsBr:Eu^2+^ NCs. Eu(OA)_3_ was reduced from Eu^3+^ to Eu^2+^ by oleylamine at 285 °C. Subsequently, CsOA and bromotrimethylsilane were injected under vigorous stirring to obtain CsBr:Eu^2+^ NCs. The resulting nanoparticles exhibited a hexagonal shape and cubic crystal structure. TEM images confirmed an average particle size of 40.0 nm (Fig. 5c). The high-resolution Eu 3d spectrum revealed four peaks: The peaks at 1164.14 eV and 1134.46 eV corresponded to Eu^2+^, while the other two peaks pertained to Eu^3+^. Analysis of the peak area ratio of Eu^2+^ and Eu^3+^ confirmed that 96.5% of Eu in the CsBr:Eu^2+^ NCs existed as Eu^2+^. The growth of CsBr:Eu^2+^ NCs could be divided into two sequential stages. In the first 0–10 min, Eu^2+^ dopants were incorporated into the CsBr host. From 10 to 30 min, the Eu^2+^ concentration within the NCs decreased with the reaction time owing to Ostwald ripening, leading to nonuniform particle sizes and the exclusion of Eu^2+^ ions from NCs. The exciton absorption intensity and PLQY increased up to 10 min, after which they decreased as the reaction progressed. This indicated that the optical properties of CsBr:Eu^2^⁺ NCs were related to the Eu^2^⁺ concentration. The CsBr:Eu^2+^ NCs exhibited an absorption peak at 340 nm (regardless of the reaction time) and displayed deep-blue fluorescence at 444 nm under 365-nm UV light, with a narrow FWHM of 30 nm. The highest PLQY of 53.4% was measured for the 10-min sample. Moreover, these NCs exhibited excitation peaks at 272, 346, and 360 nm (Fig. 5d), consistent with the findings of Yang et al. [130]. The optical properties of CsBr:Eu^2^⁺ NCs were attributable to the unique characteristics of Eu^2+^. After storage for 70 d at 25 °C and 60% relative humidity, the PL intensity decreased to 48.9% of its initial value, with no significant change in the shape of the PL peak. Additionally, when heated at 80 °C for 5 d in a glove box, the PL intensity slightly decreased to 98.6% of its initial value. These results indicate the high stability of the CsBr:Eu^2+^ NCs. An LED was fabricated using CsBr:Eu^2+^ NCs, demonstrating their potential for display applications.

In another study, a variety of Eu^2+^-doped AX NCs were synthesized to explore structural and optical variations across different host matrices [50]. Eu^2+^-doped AX NCs were fabricated using the hot-injection method. Owing to the exposed 5d orbital of Eu^2+^ ions, the emission characteristics of Eu^2+^ in alkali-metal halides were noted to be influenced by external crystal fields. This interaction facilitated the tunability of its emission spectrum, enabling efficient and stable deep-blue emission. Among AX:Eu^2+^ NCs, CsBr:Eu^2+^ NCs exhibited distinctive optical properties, demonstrating promise as highly luminescent and eco-friendly deep-blue emitters. CsBr:Eu^2+^ NCs were noted to exhibit a primitive cubic structure, where Eu^2+^ substitutes Cs^+^, forming a [EuBr_8_]^6−^ hexahedral luminescent center. The resulting CsBr:Eu^2+^ NCs presented a quasi-spherical shape with an average particle size of 38.05 ± 1.24 nm (Fig. 5e). By precisely tuning the crystal field conditions, efficient deep-blue emission could be achieved. In particular, CsBr:Eu^2+^ NCs exhibited narrow-band blue emission at 441 nm with an FWHM of 30 nm and a high PLQY of 91.1%, attributable to the hexahedron crystal-field-induced Eu-5d → Eu-4f transition (Fig. 5f). The stability of CsBr:Eu^2+^ NCs was evaluated under thermal stress, UV exposure, and environmental stressors (oxygen and moisture), and the results confirmed their strong resistance to these factors, demonstrating their reliability as emitters for LED applications. Furthermore, deep-blue LEDs based on CsBr:Eu^2+^ NCs achieved an external quantum efficiency of 3.15% and a half-lifetime of approximately 1 h (Fig. 5g and h).

## Conclusion

In this review, we systematically explored the recent advances in colloidal synthesis of Eu^2+^-based MHNCs, focusing on their unique luminescence mechanisms, synthetic strategies, and practical applications. Eu^2+^ offers narrow-band emissions, fast radiative decay lifetimes, and tunable emission wavelengths due to the parity-allowed 4f^6^5d^1^ → 4f^7^ transitions. These properties are highly sensitive to crystal field effects, coordination environments, and the host lattice structures, allowing precise tuning of emission wavelength and intensity. Among the Eu^2+^-based MHNCs discussed in this review, a redshift in emission wavelength has been experimentally observed upon halide substitution, consistent with the Dorenbos model. Specifically, CsCl:Eu^2+^ NCs exhibit an emission peak at 410 nm, whereas CsBr:Eu^2+^ NCs show emission peaks at 440 or 444 nm, indicating a redshift of more than 30 nm. Nonetheless, discrepancies in emission wavelengths have been reported even for MHNCs with identical compositions and crystal structures, suggesting that the emission properties may be influenced by various experimental factors (Table 1). Researchers have synthesized various compositions of Eu-based MHNCs via either the hot-injection or heating-up methods. Novel strategies have been proposed to significantly improve the PLQY, emission wavelength, and thermal and environmental stability. Particularly, ligand engineering, co-doping, and surface passivation strategies have proven effective in reducing non-radiative losses and enhancing luminescence performance and stability. Notably, binary or ternary MHNCs provide promising platforms for achieving deep-blue emission with narrow FWHM. We also categorized Eu^2+^-based MHNCs into two main groups: Eu^2+^ as the hosts and Eu^2+^ as the dopants, each presenting distinct challenges and opportunities. Eu^2+^-hosted structures, such as CsEuBr_3_, CsEuCl_3_, RbEu_2_Cl_5,_ Cs_3_EuCl_6_ or EuCl_2_ benefit from simplified energy transfer pathway and minimized luminescence losses. Meanwhile, Eu^2+^-doped systems—particularly in lead halide or alkali halide matrices—enable flexible bandgap engineering and demonstrate potential for integration into light-emitting diodes. Table 1 summarizes key materials, synthesis methods, optical characteristics, and potential applications. Unlike the calcination process widely used for synthesizing solid-state phosphors, colloidal synthesis—including hot-injection and heating-up methods—enable the formation of NCs at relatively low temperatures and within short reaction times. Moreover, by precisely tuning reaction parameters such as reaction temperature, reaction time, and precursors, it is possible to finely control the size, shape, crystal structure, and optical properties of the resulting NCs. When Eu^2+^ is introduced as either a hosts or a dopants in binary or ternary MHNCs, the NCs can exhibit uniform morphology, narrow size distribution, and excellent optical properties, making them highly suitable for a wide range of applications. For example, CsEuBr_3_ NCs synthesized via hot injection exhibited PLQY up to 93.51% with a narrow FWHM of 28.5 nm at 443 nm, making it a strong candidate for deep-blue light-emitting materials for WLEDs. Similarly, CsBr:Eu^2+^ NCs showed narrow-band blue emission at 441 nm with a FWHM of 30 nm and a high PLQY of 91.1%, demonstrating high performance and stability for deep-blue LEDs. Looking ahead, improving air/moisture stability and developing environmentally friendly materials remain critical challenges. To address these issues, future work must be aimed at identifying strategies to prevent the oxidation of Eu^2+^ and host NCs. Among various approaches, constructing core–shell structures has emerged as an effective approach for enhancing the stability of MHNCs and is being actively explored. Moreover, by controlling the type of surface ligands capping the MHNCs, both the stability and PLQY can be enhanced, as demonstrated in the case of lead-perovskite NCs. Moreover, the introduction of diverse divalent-cation-based hosts, such as alkaline earth or rare earth elements, can help develop promising eco-friendly alternatives for lead-free systems. Overall, Eu^2+^-based MHNCs exhibit tremendous potential for optoelectronic applications owing to their versatility, tunability, and strong luminescence. With continuous advances in materials chemistry, crystal engineering, and nanofabrication, Eu^2+^-based MHNCs are expected to play a key role in next–generation optoelectronic devices requiring bright, stable, and spectrally precise light sources.

## Data Availability

Not applicable.
